# Mathematical models use varying parameter strategies to represent paralyzed muscle force properties: a sensitivity analysis

**DOI:** 10.1186/1743-0003-2-12

**Published:** 2005-05-31

**Authors:** Laura A Frey Law, Richard K Shields

**Affiliations:** 1 Graduate Program in Physical Therapy and Rehabilitation Science, 1-252 Medical Education Bldg., The University of Iowa, Iowa City, IA, USA

## Abstract

**Background:**

Mathematical muscle models may be useful for the determination of appropriate musculoskeletal stresses that will safely maintain the integrity of muscle and bone following spinal cord injury. Several models have been proposed to represent paralyzed muscle, but there have not been any systematic comparisons of modelling approaches to better understand the relationships between model parameters and muscle contractile properties. This sensitivity analysis of simulated muscle forces using three currently available mathematical models provides insight into the differences in modelling strategies as well as any direct parameter associations with simulated muscle force properties.

**Methods:**

Three mathematical muscle models were compared: a traditional linear model with 3 parameters and two contemporary nonlinear models each with 6 parameters. Simulated muscle forces were calculated for two stimulation patterns (constant frequency and initial doublet trains) at three frequencies (5, 10, and 20 Hz). A sensitivity analysis of each model was performed by altering a single parameter through a range of 8 values, while the remaining parameters were kept at baseline values. Specific simulated force characteristics were determined for each stimulation pattern and each parameter increment. Significant parameter influences for each simulated force property were determined using ANOVA and Tukey's follow-up tests (*α *≤ 0.05), and compared to previously reported parameter definitions.

**Results:**

Each of the 3 linear model's parameters most clearly influence either simulated force magnitude or speed properties, consistent with previous parameter definitions. The nonlinear models' parameters displayed greater redundancy between force magnitude and speed properties. Further, previous parameter definitions for one of the nonlinear models were consistently supported, while the other was only partially supported by this analysis.

**Conclusion:**

These three mathematical models use substantially different strategies to represent simulated muscle force. The two contemporary nonlinear models' parameters have the least distinct associations with simulated muscle force properties, and the greatest parameter role redundancy compared to the traditional linear model.

## Background

Chronic complete spinal cord injury (SCI) induces musculoskeletal deterioration that can be life threatening. Initially muscle atrophy occurs [1], followed by muscle fiber and motor unit transformation [2-5], and ultimately lower extremity osteoporosis develops [6-10]. Maintaining paralyzed muscle tissue may prove to be a valuable means for improving the general health and well-being of individuals with SCI. Neuromuscular electrical stimulation (NMES) can be used to restore function or to impart physiologic stresses to the skeletal system in an attempt to minimize muscle atrophy and ultimately osteoporosis [11-18]. However, well-defined NMES initiated muscle forces are needed as high forces can result in bone fracture [19].

Mathematical muscle models may be essential for the determination of the necessary musculoskeletal stresses that will safely maintain the integrity of muscle and bone following SCI. Further, a clear understanding of the relationships between model parameters and muscle contractile properties or their underlying physiological processes would benefit the practical use of models for therapeutic applications. Accordingly, several approaches have been used to mathematically model electrically induced muscle forces [20-24] in able-bodied human and animal muscle.

Although muscle force production is an inherently nonlinear response of the neuromuscular system, reasonable force approximations have been achieved using linear systems [25]. A nonlinear version of a traditional 2^nd ^order system was developed by Bobet and Stein [20], and validated using cat soleus (slow) and plantaris (fast) muscle. A variation of the traditional Hill model, with additional Huxley-type modeling components (similar to the Distribution-Moment Model described by Zahalak and Ma,[26]), has evolved since its introduction [27], successfully representing submaximally activated, able-bodied, human quadriceps muscle [28-32]. While other models are available these three examples represent a diverse range of modeling approaches that allow a wide variety of discrete input patterns using constant parameter coefficients.

We are not aware of any previous comparisons of these types of models to elucidate their differences in modeling strategies. Although model parameter roles are often reported with physiologic interpretations, rarely has evidence been provided to support these physiologic (vs. mathematic) characterizations. The purpose of this study was to systematically compare one traditional linear model and two contemporary nonlinear models, using a sensitivity analysis to examine how each model's parameters influenced select simulated force properties.

The three models used different strategies to represent select force properties (peak force, force time integral, time to peak tension, half relaxation time, catch-like property, and force fusion). Further, previously reported definitions were not consistently supported by the sensitivity analyses for one of the nonlinear models. These results are important for the implementation and interpretation of future studies aimed at modeling chronically paralyzed muscle and are necessary precursors for the optimization of therapeutic stresses in attempts to maintain the integrity of paralyzed extremities and/or restore function after SCI.

## Methods

This study consists of simulated sensitivity analyses of three mathematical muscle models currently available in the literature (see below). A common, but unique, feature of each of these models is that they can accommodate inputs consisting of any number of pulses at any combination of interpulse intervals (IPIs). This input flexibility allows each model to predict a wide-range of force responses, including the impulse-response, variable or constant frequency trains, doublets, and/or randomly spaced stimulation pulses that could be useful for electrical stimulation of paralyzed human muscle.

### Linear Model

The simplest model in this study is a traditional 2^nd ^order linear model consisting of one differential equation and three constant parameters. Second order linear systems are widely used to represent a variety of dynamic systems [33] and have been used in various formats to represent muscle [25,34,35]. Although a second order linear model can be mathematically represented in several ways, the traditional linear system theory configuration was used for this analysis (1).



The parameters for this modeling strategy have well-documented mathematical definitions. Parameter *β *is the system gain, *ω*_n _is the undamped natural frequency, and *ζ *is the damping ratio (a measure of output oscillation).

Investigating the sensitivity of this traditional modeling approach for predicting simulated muscle force properties provides a valuable basis for the interpretation and comparison of more complex muscle modeling approaches, where the parameters may not be clearly defined. In addition, this model may be easily modulated with more complex feedback control systems, making clear interpretations of the parameter roles in terms of muscle force properties desirable.

### 2^nd ^Order Nonlinear Model

A nonlinear variation of a 2^nd ^order linear model was introduced by Bobet and Stein [20]. In addition to two first order differential equations (2 and 4), it includes a saturation nonlinearity (3) which saturates force at higher levels as well as a variable time constant parameter (5), which generally decreases (becomes slower) with increasing force.

*q*(*t*) = ∫exp(-*aT*)*u*(*t *- *T*)*dT *    (2)

*x*(*t*) = *q*(*t*)^*n *^/(*q*(*t*)^*n *^+ *k*^*n*^)     (3)

*F*(*t*) = *Bb *∫exp(-*bT*)*x*(*t *- *T*)*dT *    (4)

*b *= *b*_0 _(1 - *b*_1_*F*(*t*) / *B*)^2 ^    (5)

In Equation 2 the input, u(t), is a time series of the stimulation pulse train, with values of zero as the baseline and equal to 1/(delta t) at each pulse. The final output, F(t), is the modeled force over time (4), using (5) to define the variable parameter, b, as force varies over time. Parameter b varies with force based on constant parameters b_0 _and b_1_. This model has six constant parameters, B, a, b_0_, b_1_, n, and k, acting as the gain, two rate constants, and three "muscle specific constants" [20], respectively. See Table 1 for previously reported parameter definitions. Although in the original model, parameter b_1 _is constrained to values between o and 1, pilot studies using human paralyzed muscle observed better model fits when this constraint was relaxed to allow for negative values as well [36].

**Table 1:** Summary of reported parameter definitions for three mathematical muscle models.

Model	Parameter	Definition
2^nd ^Order Linear	*β *(Ns)	output gain [25, 33, 35]
	*ω*_n _(rad/s)	natural undamped frequency [25, 33, 35]
	*ζ *(-)	damping coefficient [25, 33, 35]

2^nd ^Order Nonlinear	B (N)	force gain, "maximum tetanic force" [20]
	a (1/s)	"muscle specific" rate constant [20]
	b_0_(1/s)	rate constant; maximum value of variable rate constant parameter, b, when b_1 _= zero. [20]
	b_1 _(-)	force feedback mechanism for variable rate constant, b; higher values = greater modulation of parameter b [20]
	n (-)	"muscle specific constant" used in static force saturation equation [20]
	k (-)	"muscle specific constant" used in static force saturation equation [20]

Hill-Huxley Nonlinear	A (N/ms)	Force scaling factor [21, 28, 29, 31, 32, 41, 42], and scaling factor for the muscle shortening velocity [29, 31, 41, 42]
	*τ*_1_(ms)	Force decay time constant when C_N _is absent, i.e. "in absence of strongly bound cross-bridges" [21, 28-32, 41, 42]
	*τ*_2_(ms)	Force decay time constant when C_N _is present; "extra friction due to bound cross-bridges" [21, 28-32, 41, 42]
	*τ*_c_(ms)	Time constant controlling rise and decay of C_N _[21, 28-31, 41, 42] or the transient shape of C_N _[32] and time constant controlling the duration of force enhancement due to closely spaced pulses [30]
	k_m_(-)	"Sensitivity of strongly bound cross-bridges to C_N_" [29, 31, 32, 41, 42]
	R_0_(-)	Magnitude of force enhancement due to closely-spaced pulses [28, 30] and/or from the following stimuli [29, 31, 41, 42]

### Hill Huxley Nonlinear Model

The second nonlinear mathematical muscle model has been described by its authors as an extension of the Hill modeling approach [21,27]. However, one equation in the model represents calcium kinetics not typical of Hill-based modeling approaches, and contains model components that resemble the Distribution-Moment Model [26], an extension of the Huxley model. Thus, we will use the term Hill Huxley nonlinear model to represent this modeling approach.

The most current version of this model incorporates two nonlinear differential equations, (6) and (7) [27,29-31].









Equation 6 is reported to represent the calcium kinetics involved in muscle contraction (both the release/reuptake of Ca^2+ ^as well as the binding to troponin, state variable = Cn), where variable parameter, R_*i*_, is defined in (9). R_*i *_decays as a function of each successive interpulse interval (t_*i*_-t_*i-1*_) rather than as a function of force as for the 2^nd ^order nonlinear model [27,29-31]. Equation 7 predicts force (state variable, F), based on the state variable, Cn, but has no analytical solution, requiring numerical analysis techniques to solve for force. The Hill Huxley model incorporates a total of six constant parameters, A, *τ*_1_, *τ*_c_, *τ*_2_, Ro, and km, as the gain, three time constants, a doublet parameter, and a "sensitivity" parameter [29], respectively. Please see Table 1 for previously reported parameter definitions.

### Sensitivity Analysis

**Figure 1:**
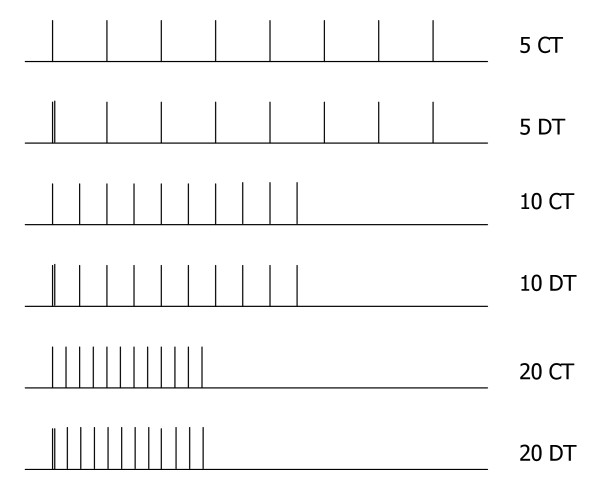
**Schematic representation of simulated force stimulation patterns**. Simulated stimulation patterns at three frequencies, 5, 10, and 20 Hz, and two types of patterns, constant train (CT) with constant interpulse intervals, and doublet train (DT) with one additional doublet pulse occurring 6 ms after the first pulse.

Simulated force trains were calculated for six different input patterns using Matlab 6.0 (Release 12, The Mathworks, Inc. USA): three constant frequency trains (CT) at 5, 10, and 20 Hz (using 8, 10, and 12 pulses, respectively), and three doublet frequency trains (DT) with base frequencies of 5, 10, and 20 Hz, but with an added pulse (doublet) 6 ms after the first pulse (using 9, 11, and 13 pulses, respectively). Please see figure 1 for a schematic representation of the input patterns.

These input patterns and frequencies were chosen to approximately correspond to a set of safe and most plausible stimulation patterns for a patient population. The risk of fracture with high frequency stimulation in individuals with SCI is considerable [19,37,38] and must be considered for the ultimate aim of validating this model for paralyzed muscle. Secondarily, to best consider parameter sensitivities at various points along the sigmoidal portion of the force frequency relationship in paralyzed muscle[39], frequencies ranging from 5 to 20 Hz were chosen in concert with 6 ms doublets (167 Hz).

The role of each parameter, in each mathematical muscle model, was determined by altering one parameter at a time, keeping all other parameters set at baseline values. The parameter increment, range, and baseline values were based on both previously reported values (Table 2) and extensive experimental pilot data (means ± 4 SD) from chronically paralyzed human soleus muscle with and without previous electrical stimulation training [36]. Previously reported parameter values varied by species [21,25,27,40] and varied through model evolutions [21,27,30,31]. Using parameter values based on pilot studies helps to provide a consistent basis necessary for between model comparisons. As no other reports of model applications in human SCI muscle were available, a wide range of values were incorporated in this study (~ +/- 4 SD of baseline) to maximize the potential for these results to be meaningful for various human paralyzed muscle applications.

**Table 2:** Parameter baselines, increments, and ranges used for the sensitivity analysis.

Model	Parameter	Range	Baseline ± Increment	Previously Reported Values	
				Human	Animal

2^nd ^Order Linear	*β *(Ns)	15 – 60	30 ± 5	0.05 – 0.5^A^	0.10 – 0.62^B^
	*ω*_n_(rad/s)	7 – 25	13 ± 2	12.6 – 18.8^A^	12.6 – 50.3^B^
	*ζ *(-)	0.4 – 1.3	0.7 ± 0.1	0.6 – 1.0^A^	1.0 – 2.0^B^

2^nd ^Order Nonlinear	B (N)	375 – 1050	600 ± 75	-	9.0 – 46^C^
	a (s^-1^)	10 – 28	16 ± 2	-	9.4 – 40^C^
	b_0 _(s^-1^)	6 – 24	12 ± 2	-	11 – 40^C^
	b_1 _(-)	-0.8 – 0.8	-0.2 ± 0.2	-	0.4 – 0.95^C^
	n (-)	1 – 10	4 ± 1	-	3.2 – 4.0^C^
	k (-)	0.1 – 1.0	0.4 ± 0.1	-	0.78 – 1.0^C^

Hill-Huxley Nonlinear	A (N/ms)	5 – 14	8 ± 1	3 – 5 ^D^	- †
	*τ*_1_(ms)	5 – 95	35 ± 10	42 – 51 ^D^	-
	*τ*_2_(ms)	30 – 165	75 ± 15	NA – 124‡ ^D^	-
	*τ*_c _(ms)	5 – 50	20 ± 5	20* ^D^	-
	k_m _(-)	0.025 – 0.25	0.1 ± 0.025	0.1 – 0.3‡ ^D^	-
	R_0 _(-)	1 –10	4 ± 1	1.14* – 2* ^D^	-

^A ^Approximate values of submaximally-activated human soleus muscle when positioned ~ neutral ankle dorsiflexion [25].

^B ^Approximate values of maximally activated cat soleus muscle [40].

^C ^Range of reported values for maximally activated cat soleus and plantaris muscle[20].

^D ^Values for submaximally-activated human quadriceps muscle in the non-fatigued state. [29, 31, 42]

† The original Hill Huxley model parameters are too different for direct comparisons [27]

* Parameter values preset at constant values.

‡ Only one representative single subject value available.

NA No reported values available in 2 of the 3 studies.

Simulated force trains were calculated for eight values of each parameter for each of the six input patterns, as well as a single twitch (for doublet analyses, see below), creating a total of 56 force profiles per model parameter. Force was simulated at 1000 Hz.

### Simulated Force Properties

For each of the CT force profiles, five specific force characteristics were determined using Matlab (Mathworks, USA): peak force (PF), defined as the maximum force at any time in the force profile; force-time integral (FTI), defined as the area under the force profile; half-relaxation time (1/2 RT), defined as the time required for force to decay from 90% to 50% of the final peak value; late relaxation time (LRT), defined as the time required for force to decay from 40% to 10% of the final peak value; and relative fusion index (RFI), defined as the mean of the last four pulses' minima divided by their succeeding four peaks (a RFI value of 1.0 indicates full fusion with no drop in force between pulses, whereas a value of 0.0 indicates no summation at all – a series of twitches reaching baseline between pulses). The time to peak tension (TPT) property, defined as the time (ms) required to reach 90% of the first peak force from time zero was determined using the 5 Hz CT pattern only. Using the DT and CT patterns at each frequency, the relative doublet PF (DPF) and doublet FTI (DFTI) were calculated. The DPF (and DFTI) were defined as the PF (FTI) of the DT and CT force differential (DT-CT) at each frequency normalized by the PF (FTI) of a single twitch. Values greater than (less than) 1.0 for either doublet property indicate more (less) force output than would be expected from a single twitch.

### Statistical Analysis

The change in each of these force characteristics with each parameter increment was calculated (7 increments for 8 parameter values) using Matlab and Excel (Microsoft Office, USA). Analysis of Variance (ANOVA) was used to determine if any parameter had a significant influence on each force property, using *α *≤ 0.05. Tukey's follow-up tests were used to determine which parameters had significant influences on each force property and relative to one another, to maintain the family wise error of 0.05 for each model.

## Results

**Figure 2:**
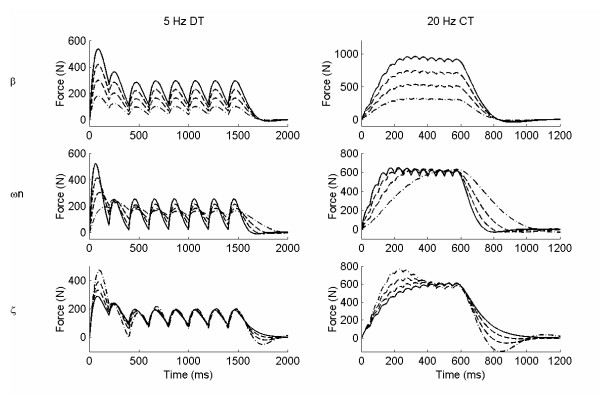
**Linear model simulated force examples**. Two simulated force trains are shown: 5 Hz doublet train, DT (left column), and 20 Hz constant train, CT (right column), with variations in each of the three individual parameter, *β*, *ω*_n _and *ζ*. Only odd numbered parameter increments are included (· -· -1^st^, - - 3^rd^, 5^th ^and – 7^th^) for clarity.

**Figure 3:**
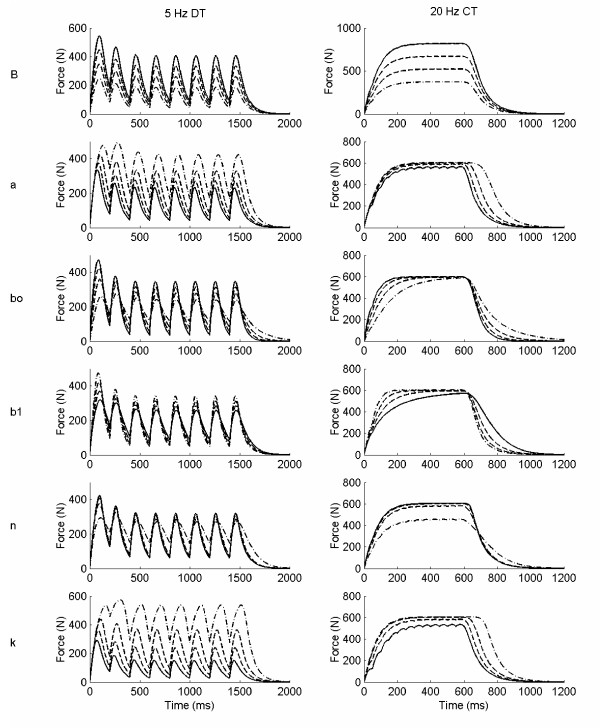
**2^nd ^order nonlinear model simulated force examples**. Two simulated force trains are shown: 5 Hz doublet train, DT (left column), and 20 Hz constant train, CT (right column), with variations in each of the six individual parameter, B, a, b_o_, b_1_, n, and k. Only odd numbered parameter increments are included (· -· -1^st^, - - 3^rd^, 5^th ^and – 7^th^) for clarity.

**Figure 4:**
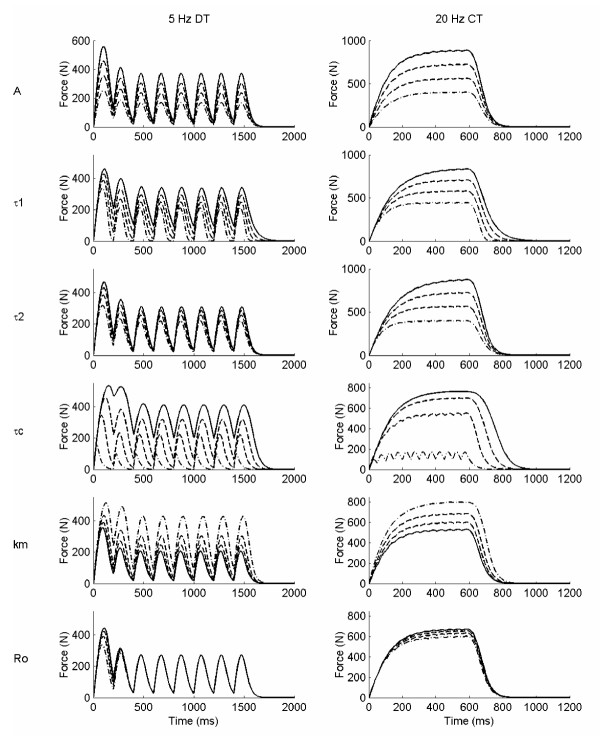
**Hill Huxley nonlinear model simulated force examples**. Two simulated force trains are shown: 5 Hz doublet train, DT (left column), and 20 Hz constant train, CT (right column), with variations in each of the six individual parameter, A, *τ*_1_, *τ*_2_, *τ*_c_, km, and Ro. Only odd numbered parameter increments are included (· -· -1^st^, - - 3^rd^, 5^th ^and – 7^th^) for clarity.

Examples of individual parameter increments on two of the six simulated force trains (5 Hz doublet train, DT, and 20 Hz constant train, CT) for the linear model, the 2^nd ^order nonlinear model, and the Hill Huxley nonlinear model are shown in figures 2, 3, and 4, respectively. The results for specific force properties are presented by model as follows.

### Linear Model

**Figure 5:**
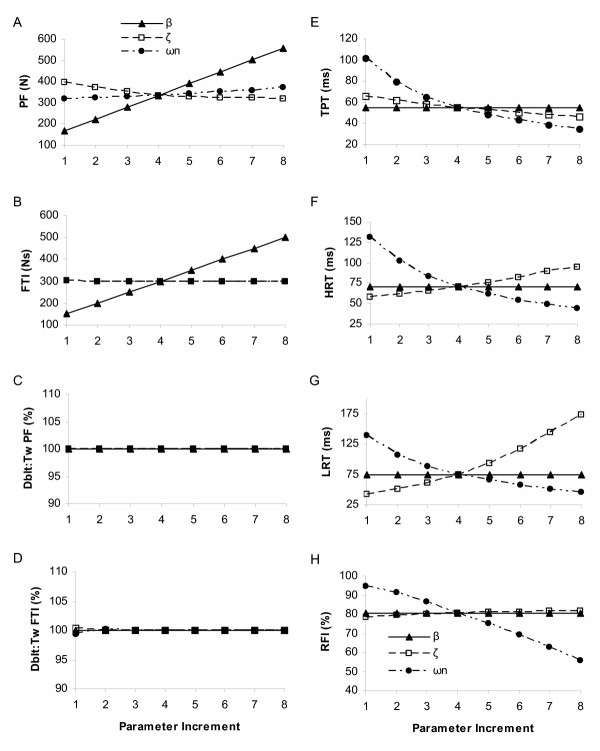
**Representation of the parameter effects on simulated force characteristics for the linear model**. Linear Model parameter effects on select force characteristics for the 10 Hz constant frequency pattern. Panel A: peak force (PF); B: force time integral (FTI); C: relative doublet PF; D: relative doublet FTI; E: time to peak tension (TPT); F: 1/2 relaxation time (HRT); G: late relaxation time (LRT); and H: relative fusion index (RFI, see text for operational definitions). Please see Table 2 for parameter baseline and increment values.

**Figure 8:**
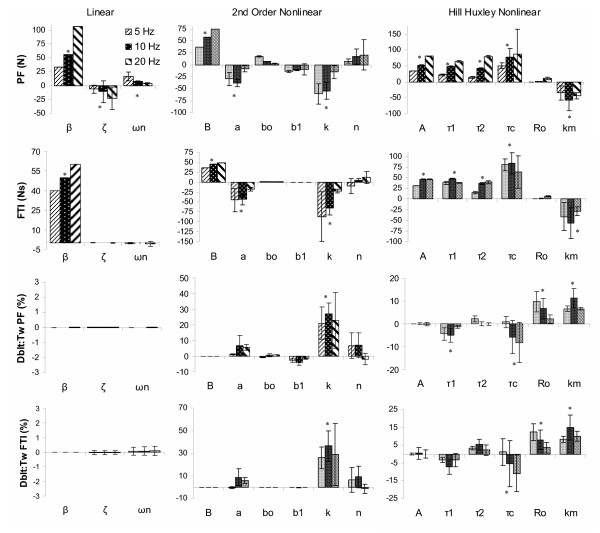
**Mean (SD) change in force magnitude characteristics per parameter increment for three muscle models**. The linear model (left column), the 2^nd ^Order Nonlinear model (middle column), and the Hill Huxley nonlinear model (right column) are shown at 5, 10, and 20 Hz. Peak force (PF) and force time integral (FTI) for the constant frequency trains (CT) are shown in rows 1 and 2, respectively. Relative doublet (Dblt) to twitch (Tw) PF and FTI, (DT-CT)/Tw, are shown in rows 3 and 4. Significant (p < 0.05) parameter influences (for 5, 10, and 20 Hz inclusive) are indicated by an asterisk (*).

The select simulated force characteristics for the three linear model parameters are shown in figure 5 using 10 Hz, consistent with the results at 5 and 20 Hz. Peak force (PF) and force time integral (FTI) were most strongly influenced at all three constant frequency trains (CT) (5, 10, and 20 Hz) by the gain parameter, *β*, with overall mean increases of 65.3 N and 50.0 Ns per 5 Ns increase in *β*, respectively (p < 0.05, figures 5 and 8), as would be expected based on previous definitions [33]. Changes in the natural frequency and the damping ratio, *ω*_n _and *ζ *respectively, produced relatively small, but significant (p < 0.05) effects on PF, but had no significant effect on FTI. No linear model parameter had any (nonlinear) effect on the doublet response relative to the twitch at any frequency (figures 5 and 8); i.e. additional pulses produced exactly the same amount of additional force a single pulse would produce in isolation, consistent with the definition of a linear system.

**Figure 9:**
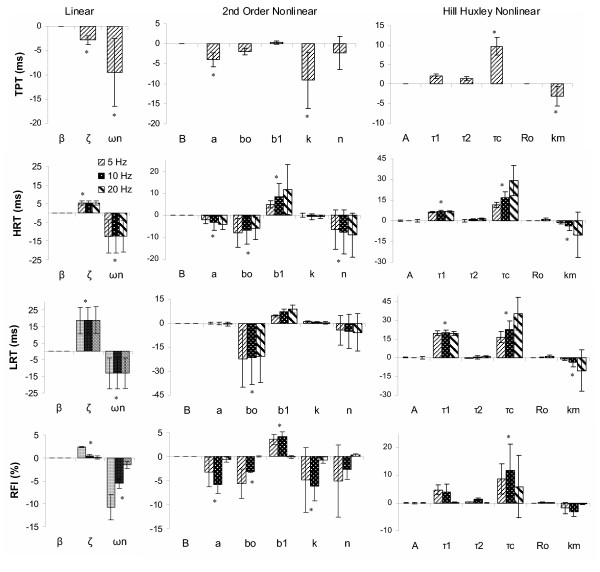
**Mean (SD) change in select force time characteristics per parameter increment for three muscle models**. The linear model (left column), the 2^nd ^Order Nonlinear model (middle column), and the Hill Huxley nonlinear model (right column) are shown. Row 1 shows the time to peak tension (TPT) for the 5 Hz constant train (CT). Rows 2, 3, and 4 show the 1/2 relaxation time (HRT), late relaxation time (LRT), and the relative fusion index (RFI), respectively, for 5, 10, and 20 Hz CTs. See text for operational definitions. Significant (p < 0.05) parameter influences (for 5, 10, and 20 Hz inclusive) are indicated by an asterisk (*).

The natural frequency, *ω*_n_, was the most influential parameter for three of the four speed properties examined as expected based on its parameter definition (Table 1): time to peak tension (TPT), half relaxation time (1/2 RT), and relative fusion index (RFI), and was a secondary influence on the late relaxation time (LRT); see figures 5 and 9. Two rad/s increments in *ω*_n _resulted in overall mean decreases of 9.6 ms, 12.5 ms, 13.1 ms, and 6.0 % for TPT, 1/2 RT, LRT, and RFI, respectively. The damping coefficient, *ζ*, also had significant (p < 0.05) influences on each force time property, but was a primary influence only for LRT, due to its strong influence on the final decay and oscillation of the system [33]. The gain parameter, *β*, had no significant effects on any of the force time characteristics, as would be expected. The simulated baseline force fusion (RFI) levels were 39.1, 80.8, and 95.3 % fused at 5, 10, and 20 Hz, respectively, indicating the simulated force baselines roughly represented a range of the force-frequency curve.

In summary, the force magnitude and force time properties were clearly divided between parameters in the linear model. Parameter *β*, the gain parameter, was the primary influence on the PF and FTI, whereas *ω*_n _and *ζ*, the natural frequency and damping ratio, were the primary and secondary influences on the four force speed properties.

### 2^nd ^Order Nonlinear Model

**Figure 6:**
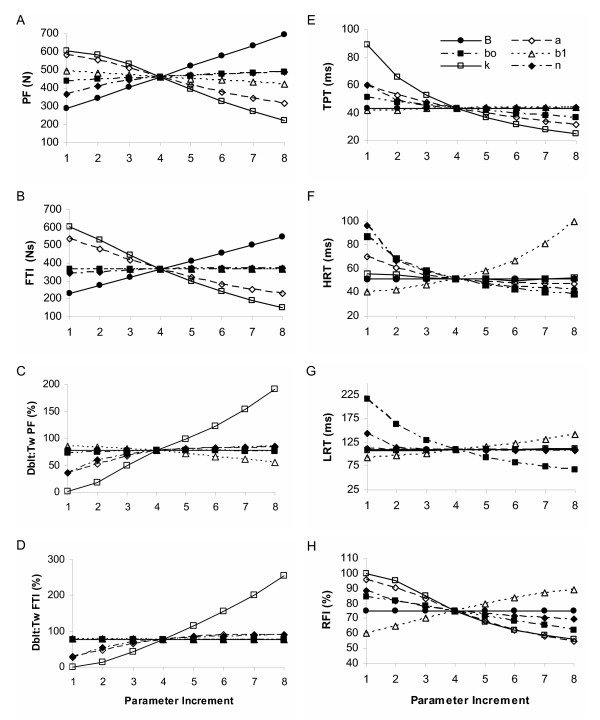
**Representation of the parameter effects on simulated force characteristics for the 2^nd ^order nonlinear model**. 2^nd ^order nonlinear model parameter effects on select force characteristics for the 10 Hz constant frequency pattern. Panel A: peak force (PF); B: force time integral (FTI); C: relative doublet PF; D: relative doublet FTI; E: time to peak tension (TPT); F: 1/2 relaxation time (HRT); G: late relaxation time (LRT); and H: relative fusion index (RFI, see text for operational definitions). Please see Table 2 for parameter baseline and increment values.

Figure 6 displays the effects of incremental changes in each of the six 2^nd ^order nonlinear model parameters on eight force characteristics using 10 Hz force trains. Similar results were found for 5 and 20 Hz. Peak force was significantly (p < 0.05) influenced by parameters B, k, and a, previously defined as the gain, a force saturation parameter and a rate constant [20]. The gain produced the greatest mean change in peak force (56.4 N per 75 N change in B, p < 0.05) followed by the saturation and rate constants, -43.2 and -25.2 N per parameter increment of 0.1 (unitless, k), and 2 s^-1 ^(a); see figures 6 and 8. Similar results were observed for the FTI, however the magnitudes of the mean FTI change per parameter increment were not different between k and B or between B and a.

The relative doublet PF and FTI (doublet trains minus constant trains, normalized by the twitch) were only significantly (p < 0.05) affected by one parameter, one of the force saturation parameters, k (figures 6 and 8, with mean changes of 23.6 % and 30.5 % for the relative DPF and DFTI per 0.1 increment in k, respectively). Thus, as k increased, the added force due to a doublet increased. However, the simulated doublet at baseline parameter values consistently produced less force than a single isolated twitch, and decreased with frequency, with added peak force values of 79.7, 76.9, and 17.9% of the twitch at 5, 10, and 20 Hz, respectively (see figure 6 for 10 Hz representation only).

There were not any direct relationships between specific parameters and force time properties for the 2^nd ^order nonlinear model. Different combinations of parameters influenced each of the force time characteristics (TPT, 1/2 RT, LRT, and RFI) (see figures 6 and 9). Consistent with Bobet's parameter definitions (Table 1), parameter b_0_, a rate constant parameter [20], most consistently influenced speed related properties overall (1/2 RT, LRT, and RFI), whereas B, the model gain, had no effect on any force time properties. The remaining four parameters, a, b_1_, n, and k, each produced significant (p < 0.05) mean changes in one or more of the force time properties evaluated (figure 9), supporting their somewhat vague previous definitions (Table 1). The force fusion (RFI) was equally influenced by parameters a, b_0_, b_1_, and k, with mean increases or decreases in force fusion ranging from 2.5 – 3.9 % per parameter increment (significant at p < 0.05). The simulated baseline force fusion levels (RFIs) encompassed a slightly wider range of the force frequency curve than observed with the linear model: 21.8, 75.3 and 99.5 % at 5, 10, and 20 Hz, respectively.

In summary, while most parameters were clearly differentiated as affecting solely force magnitude (B) or force time properties (b_0_, b_1_, and n) for the 2^nd ^order nonlinear model, this was not universally observed. Parameters k and a, a force saturation parameter and a rate constant [20], had strong influences on both force time and force magnitude characteristics, with parameter k having more primary influences (p < 0.05 per Tukey's follow-up test groupings) than any other parameter in this model. Further, more specific parameter definitions than previously provided (see Table 1) do not appear to be warranted based on this sensitivity analysis.

### Hill Huxley Nonlinear Model

**Figure 7:**
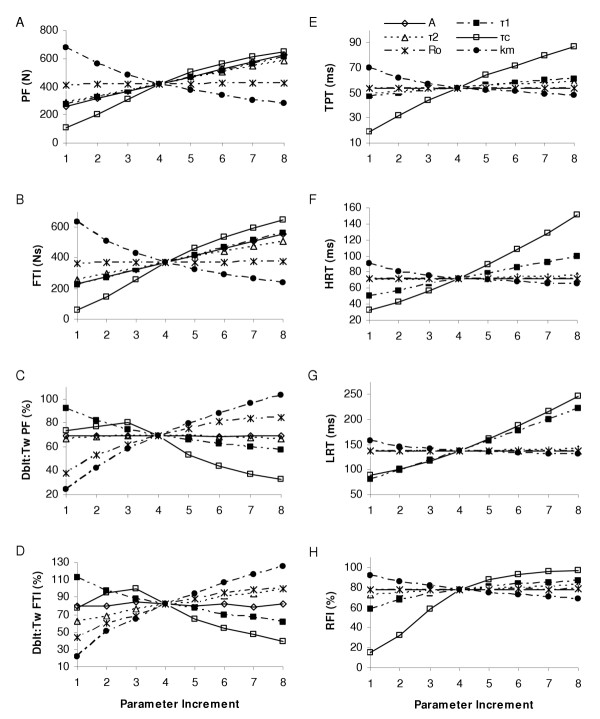
**Representation of the parameter effects on simulated force characteristics for the Hill Huxley nonlinear model**. Hill Huxley nonlinear model parameter effects on select force characteristics for the 10 Hz constant frequency pattern. Panel A: peak force (PF); B: force time integral (FTI); C: relative doublet PF; D: relative doublet FTI; E: time to peak tension (TPT); F: 1/2 relaxation time (HRT); G: late relaxation time (LRT); and H: relative fusion index (RFI, see text for operational definitions). Please see Table 2 for parameter baseline and increment values.

Figure 7 shows the effects of incremental changes in each of the six Hill Huxley nonlinear model parameters on eight force characteristics using 10 Hz force trains. Similar results were observed at 5 and 20 Hz. Peak force and FTI for the constant trains were significantly affected by five of the six parameters, but the primary influence(s) based on Tukey's groupings were a time constant parameter and the gain [29], parameters *τ*_c _and A (PF: 71.7 and 55.6 %, respectively) and *τ*_c _(FTI: 75.8 Ns). Secondary influences on PF, based on Tukey's follow-up tests, included two additional time constants and a "sensitivity" parameter [29], parameters *τ*_1_, *τ*_2_, and km, respectively (mean PF change 44.6 – 45.6 N). Secondary influences on the FTI included the gain as well: parameters A, *τ*_1_, *τ*_2_, and km (mean FTI change 30.5 – 42.1 Ns). Ro, the parameter intended to control force enhancement due to doublets [29], had no significant effect on either PF or FTI for the constant stimulation (see figure 8). The strong influences of the three time constants and the "sensitivity" parameter [29] in addition to the primary gain factor on force magnitude properties were not expected based on previous published definitions (Table 1), and suggests that one or more of these time constant parameters may play a larger role in this model than previously described.

Both the relative doublet PF and FTI (representing aspects of the "catch-like" property of muscle) were equally affected by the "sensitivity" and doublet parameters, km and Ro, (figure 8) although only the Ro parameter was specifically added to the Hill Huxley model to better represent closely spaced pulses [30]. Increments in km and Ro resulted in equivalent mean increases of 8.2 and 6.2 % for the DPF and 11.0 and 7.9 % for the DFTI, respectively (figure 8). The time constant, *τ*_c_, played a secondary role in relative doublet force with mean decreases of 4.4 and 5.1 % for doublet PF and doublet FTI, respectively (figure 8). Time constant, *τ*_1_, had an equal effect on DPF as *τ*_c_, but had no significant effect on DFTI. As with the 2^nd ^order nonlinear model, the additional peak force resulting from the simulated doublet relative to the twitch at baseline parameter values was less than 100%, and decreased with increasing frequency: 81.7, 69.4, and 26.3 % at 5, 10, and 20 Hz, respectively.

The four speed property measures were significantly influenced (p < 0.05) by three parameters in the Hill Huxley nonlinear model: time constants *τ*_c _and *τ*_1 _and "sensitivity" parameter km (figure 9), however *τ*_2 _had no significant effect on any simulated force speed property despite its previous definition (Table 1). Time constant, *τ*_c_, was consistently the primary influence (based on Tukey's follow-up tests) with mean increases of 9.7 ms, 19.2 ms, 24.8 ms, and 8.8 %, TPT, 1/2 RT, LRT, and RFI, respectively, per 5 ms increment in *τ*_c_. Secondary influences on the relaxation times (1/2 RT and LRT) included time constant, *τ*_1_, and "sensitivity" parameter, km, with mean changes of 6.7 and -5.1 ms (magnitudes not significantly different) for the 1/2 RT and 19.8 and -5.1 ms for the LRT, respectively. This finding was surprising given that in most previous publications, *τ*_c _has been kept at a constant value of 20 ms [29,30,32], and *τ*_1 _has been based on experimental late decay rates [21,29,30,32], which is not well supported by these results. Further, the only significant influence on fusion (RFI) was parameter *τ*_c_. The simulated baseline fusion levels were 10.3, 78.1, and 98.5 % at 5, 10, and 20 Hz, respectively, providing a similar range of the simulated force frequency curve as the 2^nd ^order nonlinear model.

In summary, five of the six parameters (gain, time constants, and "sensitivity") had nearly equal influences on the force magnitude properties, whereas only parameters *τ*_c_, *τ*_1_, and km (two time constant and the "sensitivity" parameter) had significant influences on force time properties, only partially supporting previously published parameter definitions. Further, parameter *τ*_c _was a primary influence for all but the doublet force characteristics.

## Discussion

A common finding between models in this sensitivity analysis was that the "gain" factors (*β*, B, and A for the linear, 2^nd ^order nonlinear, and Hill Huxley nonlinear models, respectively) each significantly altered only force magnitude characteristics, but were not the sole influence (or even the primary influence for the Hill Huxley model) on peak force. While the mathematical gain may relate to physiologic measures such as maximal tetanic force [20] or physiologic cross-sectional area, ultimately muscle force production is a result of several factors including muscle speed properties. Further, the two 2^nd ^order system models had the most clearly discernible gain parameters (*β *and B), whereas the Hill Huxley nonlinear model had equivalent gain effects from A, *τ*_1_, *τ*_2_, and km – all less than parameter *τ*_c_. Indeed, the definition of one parameter may be valid (e.g. a force gain parameter) but it is noteworthy that the parameter definition does not necessarily indicate the extent to which other parameters may also alter the physical property most commonly associated with that definition (e.g. peak force versus force gain).

Definitive physiologic force property associations were not always apparent for each model's parameters; however, parameter classifications as primarily force magnitude or force time modulators may be more appropriate. This was most clearly observed in the simple linear model, where *β *affected only force gain properties and the natural frequency, *ω*_n_, and damping ratio, *ζ*, influenced primarily the force time properties, consistent with traditional linear systems theory definitions for these parameters which have little overlap [33].

In the 2^nd ^order nonlinear model, parameters b_0_, b_1_, and a behaved primarily as rate constants and B was a pure gain factor, consistent with previous definitions (Table 1). The rate constants were not clearly differentiated by the specific force time properties commonly considered in the muscle literature (e.g. TPT and 1/2 RT), but each had varying degrees of influence on the specific speed properties. Parameters n and k from the force saturation equation in the 2^nd ^order nonlinear model played minimal and maximal roles in the model, respectively, when considering the eight force properties included in this study. Again, neither of these parameters can be easily defined physiologically, but k in particular provides a valuable contribution to the model, both due to its numerous primary influences (TPT, RFI, FTI, DPF, and DFTI) as well as its sole significant influence on the relative doublet force output.

The Hill Huxley model displayed the most parameter role redundancy with the least clearly defined individual parameter roles of the three modeling approaches. This redundancy may be beneficial for representing actual muscle forces, but it complicates the physiologic parameter interpretations often attributed to Hill-based models. Consistent with previous definitions for the Hill Huxley model parameters (Table 1), parameter A displayed purely gain characteristics, *τ*_1 _and *τ*_c _proved to be important time constants, and Ro did influence the magnitude of additional doublet force. However, *τ*_1 _was not the primary nor the sole influence on the late decay time as its definition would suggest; doublet PF and FTI were equally influenced by km and Ro, despite the definition of Ro; and *τ*_2 _had no significant effects on any force time properties contrary to expectations for a time constant. Further investigation of parameter *τ*_2_, approaching previously reported values (Table 2) in non paralyzed human muscle, further diminished the overall influence of this parameter on the force magnitude and force time properties, suggesting that the discrepancies between these results and previous definitions are not due to differences in the range investigated.

To use any of these models for experimental muscle conditions, mathematical optimization would be used to solve the underdetermined series of equations. Due to the overlapping roles of the nonlinear model parameters (figures 8 and 9), it is possible that mathematical optimization of any one parameter (and more so with multiple parameters) may alter its "physiological" meaning, as changes in one parameter can often be offset by concomitant changes in others. Although Hill- and Huxley-type models are often credited as providing physiologically meaningful parameter values [21], with the intent of using parameter values for insight into the underlying muscle contractile and fatigue mechanisms [21], this sensitivity analysis would suggest parameter values should be interpreted with caution. However, this conclusion may not extend to all nonlinear or Hill-based models, but could be the result of the many parameter substitutions and equation evolutions of this particular Hill-based model and its inclusion of Huxley-type components.

The discrepancies between the simulated parameter roles and previous definitions for the Hill Huxley nonlinear model might suggest that some previously reported parameterization techniques and assumptions may be less than ideal. Parameter *τ*_c _has been kept constant at 20 ms [21,30,31], potentially neglecting the numerous influences this parameter has on muscle force properties. The experimentally derived late decay rate has been used to estimate values for *τ*_1 _as described by Ding et al [21,30]. While this study does not directly assess the validity of this approach, it should be noted that *τ*_1 _was not the strongest influence on the late decay time. Most recently, Ro has been defined as having a linear, constant relationship with km: Ro = 1.04 + km [31]. This linear relationship was not apparent in this sensitivity study. Parameters km and Ro had similar effects on the doublet "catch-like" property of muscle, however they displayed disparate changes with increasing frequency (figure 8). Indeed, this simple linear relationship may hold true for isolated muscle conditions, such as the submaximally activated, able-bodied quadriceps muscle tested by Ding and colleagues, but possibly may not hold for human paralyzed muscle. The use of mathematical optimization techniques to determine all model parameters may circumvent the dependence of the model on potentially erroneous or incomplete parameter definitions. This optimization approach has been used for both 2^nd ^order models [20,25,40].

The linear, individual investigation of parameter sensitivities is a potential limitation of this study. Particularly for the nonlinear models, interactions between parameters are likely to exist, which may not be fully exhibited within these results. However, altering multiple parameters at a time, while in theory useful, could produce highly complex results, making study assessments practically infeasible. This systematic sensitivity analysis approach provides valuable information regarding the different parameters' influences on force characteristics and illuminates each model's approach to mathematically representing physiologic phenomena that has not been previously investigated. Clinical scientists in rehabilitation must continue to understand the meaning of various muscle models in an effort to develop effective therapeutic interventions. This sensitivity analysis provides a framework for investigators to compare and choose a model that is most appropriate for the clinical application.

## Conclusion

The key findings of this study were 1) the linear model parameters were clearly separated between simulated muscle force gain and speed properties, whereas this delineation was blurred for the two nonlinear models; 2) simulated force magnitude (PF) was generally influenced by multiple parameters for the nonlinear models, not solely by the defined force gain factors; 3) the reported physiologic parameter definitions were not consistently supported by the results for the Hill Huxley nonlinear model; and 4) these three mathematical models utilize substantially different approaches for representing muscle force, as indicated by the differences in parameter roles observed for each model.

This sensitivity analysis provides a strong framework to better understand the roles and sensitivities of each parameter for three mathematical muscle models as well as a means to compare their different modeling strategies. The results of this study will help researchers better understand the similarities and differences among three possible modeling approaches, assist in the interpretation of parameter values with varying muscle conditions (e.g. fatigue or contractile protein adaptations), and may provide valuable information necessary for choosing the most appropriate modeling approach for a particular application. The three models evaluated each use constant parameters to modulate their force outputs; given the same inputs these results conclude that they employ notably different strategies using constant parameters that do not consistently match previously reported definitions (Hill Huxley nonlinear model in particular). Further experimental studies will be needed to assess which model is best suited for use with human paralyzed muscle applications.

## Abbreviation List

CT constant frequency trains

DT doublet frequency trains (single doublet at the start of a CT)

DPF doublet peak force normalized by twitch peak force

DFTI doublet force time integral normalized by twitch force time integral

FTI force time integral

1/2 RT half relaxation time

Hz Hertz

LRT late relaxation time

N Newtons

PF peak force

RFI relative fusion index

s seconds

TPT time to peak tension

## Competing interests

The author(s) declare that they have no competing interests.

## Authors' contributions

LAFL carried out all force simulations and calculations, performed statistical analysis and drafted the manuscript. RKS participated in the design and coordination of the study and critical revisions of the manuscript. Both authors read and approved of the final manuscript.
